# Identification of Associated SSR Markers for Yield Component and Fiber Quality Traits Based on Frame Map and Upland Cotton Collections

**DOI:** 10.1371/journal.pone.0118073

**Published:** 2015-01-30

**Authors:** Hongde Qin, Min Chen, Xianda Yi, Shu Bie, Cheng Zhang, Youchang Zhang, Jiayang Lan, Yanyan Meng, Youlu Yuan, Chunhai Jiao

**Affiliations:** 1 Institute of Cash Crops, Hubei Academy of Agricultural Sciences, Wuhan, China; 2 Key Laboratory of Cotton Biology and Breeding in the Middle Reaches of the Changjiang River (Wuhan), Ministry of Agriculture, Wuhan, P. R. China; 3 Institute of Cotton Research, Chinese Academy of Agricultural Sciences, Anyan, China; 4 Hubei Academy of Agricultural Sciences, Wuhan, China; USDA-ARS-SRRC, UNITED STATES

## Abstract

Detecting QTLs (quantitative trait loci) that enhance cotton yield and fiber quality traits and accelerate breeding has been the focus of many cotton breeders. In the present study, 359 SSR (simple sequence repeat) markers were used for the association mapping of 241 Upland cotton collections. A total of 333 markers, representing 733 polymorphic loci, were detected. The average linkage disequilibrium (LD) decay distances were 8.58 cM (r^2^ > 0.1) and 5.76 cM (r^2^ > 0.2). 241 collections were arranged into two subgroups using STRUCTURE software. Mixed linear modeling (MLM) methods (with population structure (Q) and relative kinship matrix (K)) were applied to analyze four phenotypic datasets obtained from four environments (two different locations and two years). Forty-six markers associated with the number of bolls per plant (NB), boll weight (BW), lint percentage (LP), fiber length (FL), fiber strength (FS) and fiber micornaire value (FM) were repeatedly detected in at least two environments. Of 46 associated markers, 32 were identified as new association markers, and 14 had been previously reported in the literature. Nine association markers were near QTLs (at a distance of less than 1–2 LD decay on the reference map) that had been previously described. These results provide new useful markers for marker-assisted selection in breeding programs and new insights for understanding the genetic basis of Upland cotton yields and fiber quality traits at the whole-genome level.

## Introduction

Cotton is an important industrial crop in China. Many cotton breeders have focused on detecting and using marker-associated quantitative trait loci (QTLs) for marker-assisted selection (MAS) in breeding programs. Linkage analysis is a classic strategy for detecting QTLs in segregated populations derived from two inbred lines. Since Shappley [1] first reported QTLs associated with the agronomic and fiber traits of Upland cotton, thousands of QTLs have been identified through segregation analyses in cotton [2–15]. Two population types have been used in these QTL mapping studies: populations derived from interspecies crosses between *Gossypium hirsutum* and *Gossypium barbadense* and populations derived from intraspecies crosses within *G*. *hirsutum*. Most QTLs and linkage markers detected based on these interspecies populations are difficult to directly utilize for MAS because the Upland cotton varieties or lines are major material resources in breeding programs. However, when QTL and linkage markers were detected in intraspecies populations, only a few genomic areas can be scanned because of the low number of polymorphisms between Upland cotton varieties, and these results are only suitable for breeding populations derived from QTL-detected populations. For better understand the genetic basis of interesting traits in different breeding materials, such as the QTL distributions, configurations, and the percentage contribution to phenotypic variation, cotton breeders need to employ new analysis method.

In recent years, association mapping based on disequilibrium analysis has been introduced into plant QTL mapping. The new mapping strategy provided a powerful method for QTL mapping of germplasm populations. Compared with QTL mapping based on linkage analysis, association mapping has many advantages, including a higher resolution, increased genome coverage, lower time and money consumption, and reduced risk. Abdurakhmonov et al. [16] first conducted association mapping in which association between SSR (simple sequence repeat) markers and fiber quality traits was detected based on a germplasm resource population comprising 208 landrace stocks and 77 photoperiodic variety accessions, and a core set of 95 microsatellite markers. Abdurakhmonov et al. [17] also conducted genome-wide linkage disequilibrium (LD) scanning and association mapping based on a panel consisting of 334 *G*. *hirsutum* variety accessions from Uzbek, Latin American, and Australian ecotypes. In two environments, an average of 20 SSR markers were found to be associated with the main fiber quality traits using a unified mixed liner model (MLM) incorporating population structure and kinship, and 12–22 SSR markers were associated with fiber length, fiber strength, fiber fineness and six other fiber quality traits. Approximately 25% to 54% of these markers had previously been detected in studies based on linkage analysis. Zeng et al. [18] identified associations between SSR markers and fiber traits using an exotic germplasm population derived from species polycrosses (SPs) among tetraploid *Gossypium* species. A total of 202 fragments were analyzed, and fifty-nine markers showed a significant association with six fiber quality traits. These studies confirmed the feasibility of applying association analysis to explore complex traits in Upland cotton collections.

Following system and cross selection, the Upland cotton varieties found in China were demonstrated to show distinct characteristics. Generally, Chinese Upland cotton varieties are typically classified into three ecotypes: the Yellow River valley type, the Yangtze River valley type and the interior land type, according to the areas in which cotton was planted and cultivated. The Yellow River valley type is characterized by high disease resistance and high yields, while the Yangtze River valley type exhibits a high lint percentage or large bolls. Additionally, the interior land type shows adaptation to long days and short growing seasons in high-latitude areas. Furthermore, a large number of germplasm resources, including high lint percent and fiber quality lines, have been developed through cotton breeding. These varieties and germplasm resource lines have provided important materials for improving the yields and fiber quality of Upland cotton varieties in China. Zhang et al. [19] performed general linear model (GLM) association mapping of 12 agronomic and fiber quality traits based on 121 SSR markers and 81 *G*. *hirsutum* L. collections, and detected 180 loci that were significantly associated with 12 traits in more than one environment. Mei et al. [20] conducted association mapping of yields and yield component traits using 356 representative Upland cotton cultivars and 145 polymorphism markers. Cai et al. [21] performed association mapping of fiber quality traits in 99 *G*. *hirsutum* L. collections with 97 polymorphic microsatellite marker primer pairs. Zhao et al. [22] carried out association mapping based on *Verticillium* Wilt Resistance using a collection of 329 cotton (*G*. *hirsutum* L.) accessions obtained from a Chinese cotton germplasm collection. The results of these studies indicated the feasibility of applying association analysis to explore complex traits in Upland cotton collections in China. To better understand the genetic foundation of the yield and fiber quality traits at the population level and identify associated SSR markers, we performed whole-genome association analyses using 359 SSR polymorphism markers well distributed in reference maps [23, 24] and a panel of 241 varieties and germplasm resource lines in the present study.

## Materials and Methods

### Selection of accessions and determination of phenotypic data

A total of 241 Upland cotton accessions were selected for genotype screening and evaluation of yield components and fiber quality traits to identify loci associated with yield components and fiber quality QTLs. All of the collections were derived from four sources: ① elite varieties popularly cultivated in China; ② germplasm resource lines with outstanding yield components or fiber qualities; ③ parental lines that are typically used in breeding programs; and ④ historical varieties and germplasm resources lines from abroad, including 20 collections from the US, 6 from the Uzbek, 6 from the Sudan, one from Australia and one from Cuba (S1 Table). All of the materials are available from Institute of Cash Crops, Hubei Academy of Agricultural Sciences (ICC-HBAAS) and the National Mid-term Genebank of the Institute of Cotton Research, Chinese Academy of Agricultural Sciences (ICR-CAAS) after signing a Material Transfer Agreement (MTA).

Phenotypic data were obtained in 2010 and 2011 from two locations in Hubei Province, China with different climates: (1) Wuhan Breeding Station (N30°28′54″, E114°18′30″), Hubei Academy of Agricultural Sciences (HBAAS), Wuhan city (in the east of Hubei Province) and (2) Institute of Agricultural Sciences, (N30°25′08″, E112°47′43″, in the middle of Hubei Province), Qianjiang city, Hubei Province. Field planting was approved by HBAAS. No specific permissions were required for these locations/activities. The field studies did not involve endangered or protected species. A complete randomized block design with three replications was employed for each location every year. For statistical analysis, the locations and years were treated as factors of different environments: environment 1 = Wuhan in 2010; environment 2 = Qianjiang in 2010; environment 3 = Wuhan in 2011; and environment 4 = Qianjiang in 2011. The plot size was 0.8 m wide and 5.0 m long, with thirteen individuals per replication and a plant density of 33,000 plants ha^-1^. The measurements of each yield component trait and fiber quality trait obtained from the 241 collections were averaged over three replicates. The following yield component traits were evaluated: the number of bolls per plant (NB), boll weight (BW), and lint percentage (LP). Ten plants growing near to each other were selected to count the total number of bolls. The average number for ten plants was scored as NB. Twenty-five bolls from each plot were weighed to determine the BW and then ginned by roller gin to evaluate the LP. Fifteen grams of lint was sent to the Supervision and Testing Center of cotton quality, the Ministry of Agriculture to measure the fiber quality. The following fiber quality traits were evaluated using the High-Volume Index (HVI) spectrum: 2.5% fiber span length (FL, mm), fiber strength (FS, cN/tex), and the micronaire reading (FM).

### SSR markers and genotyping

In 2010, the young, not yet fully expanded leaves were collected from five plants of each line. DNA was extracted from the leaves as previously described [25]. A total of 359 polymorphic SSRs were used to genotype the 241 Upland cotton collections. The 359 SSRs included three resources: ① 302 SSRs separated by a distance of approximately 10.0 cM on all 26 chromosomes (A1–A13, D1–D13), and covered 94.6% (3241.3 cM/3425.8 cM) of the reference map [23, 24]; ② 27 markers separated by approximately 1–3 cM distance on the 50.0–80.0 cM area of A1 and D1 chromosomes; and ③ 30 markers linked to the QTLs of three yield components traits (NB, BW, and LP) and three fiber quality traits (FL, FS, and FM). The SSR primer sequences used in these analyses were obtained from the Cotton Microsatellite Database (CMD, http://www.cottonmarker.org/). The marker nomenclature consisted of a letter that specified the origin of the marker followed by the primer number. As previously described [26], the SSR analysis was conducted by polymerase chain reaction (PCR) and 6% non-denaturing polyacrylamide gel electrophoresis (PAGE). PCR runs were performed for 30 cycles of 45 s at 94°C at the annealing temperature for 45 s and 72°C for 60 s, and a final extension step at 72°C for 5 min. For each SSR primer, the polymorphic bands were identified according to the fragment size. The presence of polymorphic DNA fragments was scored as 1, and the absence of fragments was scored as zero. Multiple polymorphic DNA fragments presented or absented together in panel were identified as same marker locus. For the STRUCTURE software, “1” indicates fragments present, “0” indicates absent, and “-1” indicates missing data. For the Tassel software, “2/2” indicates fragments present, “1/1” indicates absent, and “0/0” indicates missing data.

### Data analysis

LD values (r^2^ and *p* value) between marker fragments were calculated using TASSEL 3.0 software [27]. The genetic distances between marker pairs were calculated based on the position of these markers on the genetic map [23, 24]. Minor loci with a frequency < 0.05 were filtered out to reduce problematic and biased LD estimations between pairs of loci [28, 29]. The r^2^ values for pairs of SSR loci were plotted as a function of map distances, and LD decay (r^2^ < 0.1) was estimated using the average distances of marker pairs showing LD values lower than 0.1 [30].

Analysis of variance (ANOVA) for the phenotypic data was conducted using the Statistical Analysis System (SAS8.1, Cary, NC). The broad-sense heredity of the six traits was calculated using the following equation:

$$H_{2}=\sigma_{G}^{2}/(\sigma {}_{G}^{2}+\sigma_{e}^{2})$$

where ***σ*** ^2^_*e*_ is the residual variance component, and ***σ*** ^2^_*G*_ is the genotypic variance component. The population structure was analyzed using STURCTURE 2.2 software [31, 32, 33], with a running time of 100,000 and 50,000 replications after burn-in. Models for admixture and correlated allele frequencies were employed in the population structure analysis. The pairwise kinship of all 241 collections was calculated using TASSEL 3.0 software [27]. The MLM association analysis of the yield components and fiber quality traits was performed with TASSEL 3.0 software, incorporating filtered marker data and the K and Q matrices. We also performed GLM association analyses using the same four datasets, incorporating pairwise kinship information as a covariate and 1,000 permutations for the correction of multiple testing. To make up for the deficiency of using *p*-values in association, significant MLM associations (*p* < 0.05) across more than two environments were ranked, and the significance of these markers (*p* < 0.05) in the permutation test was compared using GLM association tests. The *p*-values derived from the MLM and GLM analyses were also separately tested using the positive false discovery rate (pFDR) test [34] for multiple testing corrections. The minimum Bayes factor (BFmin) was calculated using following formula: BFmin = -e*p*ln(p) [17, 35, 36].

## Results

### Amplification fragment polymorphisms of the SSR markers

A total of 359 SSR markers were used to genotype 241 collections, among which 26 (7.2%) of the markers presented homomorphisms, and 333 markers covering 86.6% reference map (2968 cM/3425.8 cM) produced 733 polymorphic loci, averaging 2.2 loci per marker. The observed locus frequencies ranged from 50.19% to 99.62%, averaging 78.17%. The average genetic diversity was 0.358 (ranging from 0.008 to 0.802). The average polymorphism information content (PIC) was 0.300 (ranging from 0.008 to 0.773).

### Population structure and LD of the marker pairs

**Fig 1:**
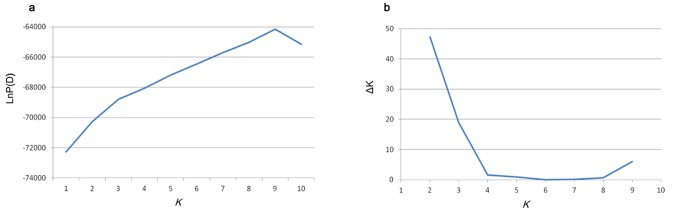
Estimated LnP(D) and Δ*K* from 10 iterations obtained through STRUCTURE analysis. (a) LnP(D) for k values from 1 to 10 for simulations using all 241 collections. (b) ΔK for k values from 2 to 9 for all 241 collections.

The population structure was determined using STRUCTURE software, with K values ranging from 1–10. The LnP(D) value increased continuously with no obvious inflexion point before the panel was divided into 9 subgroups. However, the Δk value decreased rapidly at K = 2 and K = 3, and the locus frequency divergence among the subpopulations (Net nucleotide distance) was significant at k = 2, but not at k = 3. Fig. 1 shows that Δk presented a second peak for K = 9, indicating that this panel could be continuously further divided until into 9 subgroups. Pritchard et al. [31] suggested focusing on values of K that capture most of the structure in the data and that seem biologically sensible when the model choice criterion continues to increase with increasing K. To avoid an overcorrected population structure that would lead to the disappearance of the association loci in the association analysis [37], we adopted K = 2, not 9. The first subgroup contained 120 collections, comprising the majority of the elite varieties and parental lines that are typically used in breeding programs from the Yangzi river valley. The second subgroup included 121 collections consisting of germplasm resources lines, historical varieties from abroad, and the majority of the elite varieties and parental lines from the Yellow river valley (S1 Table). Cluster analysis of 241 Upland cotton collections showed majority of subgroup 1, as well as majority of subgroup 2, was clustered together (S1 Fig.).

**Fig 2:**
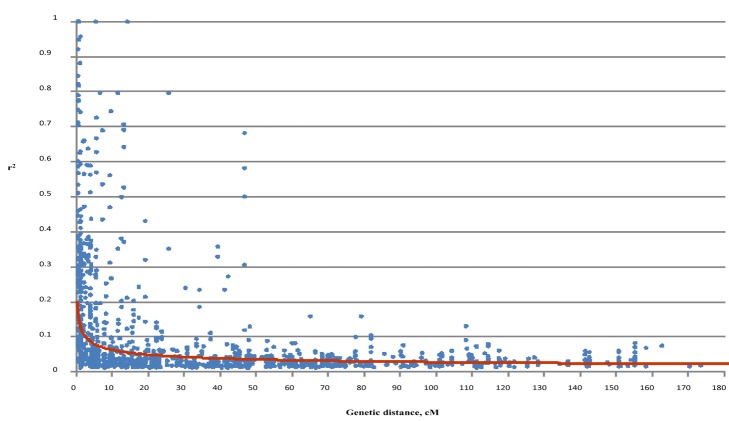
Linkage disequilibrium (LD) decay according to genetic distance (cM). LD decay is considered present when r^2^ < 0.1

Approximately 9.36% of the marker pairs showed significant LD, with *p* values lower than 0.05 (S2 Table). Approximately 18.90% of the collinear marker pairs showed significant LD, and 40.50% of the obtained LD values (r^2^) were greater than 0.1. Approximately 8.87% of the non-collinear marker pairs showed significant LD, and 3.45% of the LD values (r^2^) were greater than 0.1. Most of the significant LD values were higher than 0.2 were obtained from collinear marker pairs (S2 Fig., S2 Table). The LD value (r^2^) decreased rapidly at genetic distances of less than 10 cM. The longest genetic distance between markers was 108 cM. The average genetic distance between markers was 8.58 cM and 5.76 cM for r^2^ > 0.1 and r^2^ > 0.2 (Fig. 2).

### Performance of phenotype and Broad-sense heritability

For all yield and fiber quality traits, the 241 collections presented a wide-range of phenotypic variation in the four different environments (S3 Table). For example, in environment 1 (E1), the NB, BW and LP ranged from 6.49 to 26.75, 2.93 to 5.47 and 28.22% to 45.0%, respectively, and the FL, FS, FM ranged from 25.21 to 33.33, 24.27 to 35.40 and 3.94 to 5.64. Analysis of ANOVA against different locations (Wuhan, Qianjian) and different years (2010, 2011) revealed that all six traits were significantly influenced by different environments, except for LP by location (Table 1).

**Table 1:** Mean squares of the ANOVA for yield and fiber quality traits of 241 collections across two years and two locations.

Source	df	NB	BW	LP	FL	FS	FM
Collection	240	31.18*	0.63***	30.14***	4.55***	6.15***	0.44***
Year	1	9752.10***	82.26***	172.27***	14.20***	9.43*	26.73***
Location	1	217.08**	7.90***	0.18	199.14***	17.02***	54.93***
Error	721	24.76	0.42	2.39	1.42	1.51	0.27

*Significant at the *p* < 0.01 level,

**Significant at the *p* < 0.001 level,

***Significant at the *p* < 0.0001 level.

Among the six evaluated traits, LP showed the highest broad-sense heritability, ranging from 0.67 to 0.81. FS and FL exhibited heritabilities higher than 0.5 in three environments and lower than 0.5 in one environment. The broad-sense heritability of the FM was lower than 0.5 in three environments and higher than 0.5 in one environment. The NB and BW showed lower heritabilities compared with the other traits across the four environments, ranging from 0.33–0.42 (Table 2, S4 Table).

**Table 2:** Broad-sense heritability of the six traits in four environments.

Traits	E1	E2	E3	E4
NB	0.38	0.33	0.42	0.35
BW	0.40	0.42	0.38	0.41
LP	0.67	0.71	0.79	0.81
FL	0.40	0.55	0.59	0.51
FS	0.42	0.53	0.56	0.54
FM	0.41	0.42	0.67	0.47

### Association mapping

For all six traits, including the three yield component traits (NB, BW and LP) and the three fiber quality traits (FL, FS and FM), we applied an MLM (+ kinship + Q-matrix) model to analyze the four datasets derived from the 241 collections at two locations over two years. Only markers showing significance in more than one environment were used to further test significance through the FDR and BFmin. To compare the results of the GLM and MLM, we also used a GLM (+ kinship) model to analyze the four datasets and conduct permutation testing.

Twenty markers tolerated the FDR test in one or more environments, including 12 for yield traits and 8 for fiber quality traits. Fifty one marker loci (25 for yield traits and 26 for fiber quality traits) presented moderate-to-strong or strong-to-very strong evidence for association in different environments. Sixteen markers for yield traits and nine for fiber quality traits passed the permutation test at the 0.05 level in the GLM analysis. In total, forty-six markers associated with different traits were accepted in our analysis (Table 3 and Table 4).

**Table 3:** SSR markers associated with the same yield component traits in different environments using the MLM method.

Trait	Marker	Chr.	GLM^a^	P1^b^	P2^b^	P3 ^b^	P4 ^b^
NB	BNL3261	A12	4	0.0011^m^ ^F^ ^P^			0.0006^M^ ^F^ ^P^
	BNL3590	D3	4		0.0077^m^ ^f^		0.0009^M^ ^F^ ^P^
	NAU2581	UL	3			0.0000^M^ ^F^ ^P^	0.0037^m^ ^f^
	NAU3522	A13	3	0.0013^m^ ^F^ ^P^		0.0009^M^ ^F^ ^P^	
	cgr6807+	D1	2	0.0037^m^ ^f^			0.0029^m^ ^P^
	cgr6356	A1	2	0.0043^m^ ^f^ ^0^	0.0057 ^m^ ^f^		
	NAU2985	D1	4		0.0065^m^ ^f^	0.0161^f^	0.0030^m^ ^f^
	NAU3861	D13	2	0.0381^f^		0.0075^m^ ^f^	0.0098^m^ ^f^
BW	dPL0542	D1	3	0.0440		0.0005^M^ ^F^ ^P^	
	NAU1190	A3	2	0.0425	0.0011^m^ ^F^		0.0469
	NAU2859	D3	2		0.0470	0.0003^M^ ^F^	
LP	CIR307	D1	4	0.0360^f^	0.0062^m^ ^f^ ^P^		
	BNL3590* +	A2	4		0.0096^m^ ^P^	0.0148^m^ ^f^	
	JESPR197	A5	4			0.0011^m^ ^F^ ^P^	0.0036^m^ ^f^
	NAU2581	UL	3			0.0032^m^ ^f^ ^P^	0.0227^f^
	NAU3053	D7	2	0.0002^M^ ^F^	0.0004^M^ ^F^	0.0037^m^ ^f^	0.0039^m^ ^f^
	NAU3206	A6	3	0.0147^f^	0.0028^m^ ^f^ ^P^	0.0193^f^	0.0030^m^ ^f^ ^P^
	NAU3293	D12	4	0.0216^f^		0.0008^M^ ^F^ ^P^	0.0114^f^
	NAU3308* +	D2	2			0.0050 ^m^ ^f^	0.0079^m^ ^f^
	NAU3522	A13	2	0.0217^f^	0.0483		0.0021^m^ ^f^ ^P^
	NAU3778	A12	2	0.0017^m^ ^F^			0.0186^f^
	NAU3995+	A3	2			0.0169^f^	0.0018^m^ ^F^
	BNL1395* +	D7	4	0.0346^f^ ^P^	0.0397^f^ ^P^		
	JESPR204*	D13	3		0.0193^f^ ^P^	0.0239^f^	
	NAU862	A3	4	0.0105^f^ ^P^	0.0426		
	BNL1672*	A9	4	0.0099^m^ ^f^		0.0233^f^	
	BNL1705	D11	4	0.0099^m^ ^f^	0.0020^m^ ^f^		
	NAU2251	A12	2	0.0048^m^ ^f^	0.0267^f^		

* Linked or associated with the same traits in previous reports;

^**+**^ Separated from markers that are linked or associated with the same traits described in previous reports at distances of less than 1–2 LD decay cM on the reference map;

^**a**^ times repeatedly detected using GLM;

^**b**^ *p* value in the E1, E2, E3 and E4 environments;

^**M**^ BF_min_ strong-to-very strong evidence for association (*p* < 0.05);

^**m**^ BF_min_ moderate-to-strong evidence for association (*p* > 0.05–0.13);

^**P**^ significant in the GLM test after 1,000 permutations at *p* < 0.05;

^**F**^ significant in the pFDR MLM test at *p* < 0.05;

^**f**^ significant in the pFDR GLM test at *p* < 0.05.The values formatted in bold were not supported by any evidence other than *p* value (*p* < 0.05).

**Table 4:** SSR markers associated with the same fiber quality traits in different environments using the MLM method.

Traits	Markers	Chr.	GLM ^a^	P1 ^b^	P2 ^b^	P3 ^b^	P4 ^b^
FL	BNL1395*	D7	4	0.0046^mfP^		0.0142^f^	0.0104^f^
	DC40182*	UL	4	0.0007^MFP^	0.0003^MFP^	0.0011^mFP^	0.0004^MFP^
	NAU2980*	D13	2	0.0312^f^	0.0011^mF^		0.0460
	NAU2641	D6	4	0.0240^f^	0.0115^fP^	0.0258^f^	0.0231^f^
	NAU2776	D10	4	0.0414		0.0332^f^	0.0111^fP^
	NAU3455	D8	4	0.0444	0.0204^fP^		
	NAU3881	D12	4	0.0456			0.0366^fP^
	BNL2572*	A4	2	0.0358	0.0057^mf^		0.0217^f^
	BNL3594	D6	2		0.0276^f^	0.0031^mf^	
	CIR307+	D1	2	0.0052^f^	0.0168^f^		**0.0440** ^f^
	NAU2723	A9	2	0.0419			0.0067^mf^
	NAU3110	D5	4	0.0250^f^		0.0037^mf^	0.0038^mf^
FS	JESPR153*	D13	3	0.0283^f^	0.0007^MF^		0.0047^m^
	NAU3736+	D1	2	0.0012^mF^	0.0011^mF^		
	NAU3778	A12	2	0.0005^MF^	0.0477	0.0125^f^	0.0461
	NAU5411	A1	2	0.0017^m F^	0.0061^mf^		
	BNL3594	D6	2	0.0047^mf^		0.0364^f^	
	BNL827	D6	2			0.0251^f^	0.0072^mf^
	CIR307*	D1	2	0.0121^f^	0.0080^mf^		
	DC40182*	UL	4	0.0283^f^	0.0031^mf^		0.0192^f^
	NAU2894	D5	2	0.0176^f^	0.0055^mf^		
	NAU3110	D5	4	0.0236^f^	0.0065^mf^	0.0058^mf^	0.0341^f^
	NAU3995	A3	2	0.0058^mf^	0.0342^f^		
	TMB1618*	UL	2	0.0326^f^	0.0023^mf^		
FM	JESPR274*	A9	4	0.0017^mF^		0.0344^f^	
	NAU3700+	D3	2		0.0003^MFP^	0.0278^f^	
	JESPR101+	D3	2	0.0146^P^	0.0187^P^		
	NAU3881	D12	4	0.0285^f^	0.0100^mP^		0.0383^f^
	NAU2723	A9	2			0.0029^mf^	0.0297^f^
	NAU3703	A11	2	0.0027^mf^		0.0041^mf^	
	NAU5508	D9	2	0.0041^mf^		0.0068^mf^	

* ^**+ a, b, f, F, m, M, P**^have the same meanings as described in Table 3

Of the 46 association markers detected across more than one environment, 8 were associated with NB, 3 were associated with BW, 17 were associated with LP, 12 were associated with FL, 12 were associated with FS, and 7 were associated with FM (Tables 2 and 3). Among these 46 SSRs, SSR CIR307/D1 was associated with three traits; 12 SSRs (NAU3995/A3, NAU2723/A9, NAU3522/A13, CIR307/D1, cgr6807/D1, BNL3590/D3, NAU3110/D5, BNL3594/D6, BNL1395/D7, NAU3881/D12, DC40182 and NAU2581) were associated with two traits; and the remaining 34 SSRs were each associated with one trait.

We compared the associated markers identified in the present study with SSR markers previously identified through linkage QTL and association mapping analyses [2–22, 39–43]. Among 46 markers, 14 were found to be associated or linked with the same traits (LP, FL, FS and FM) identified in previous studies (Table 5). Of the 14 markers, five were associated with LP, four were associated with FL and FS respectively, and one was associated with FM. Because the different markers were used in different studies, only a few markers could be directly compared. Therefore, we also employed the reference map as a bridge to compare the results obtained in the present study with the results from previous studies [2–22, 39–43]. Nine markers were found to be near the QTLs controlling the same traits with a distance of less than 1–2 LD decay on the reference map (Table 6).

**Table 5:** Markers associated or linked with the same traits in the present study and previous studies.

Trait	Markers	QTL	References
LP	BNL3590	TC-qLP-c2–1	15, 20^A^
	BNL1395	qLP-16–1	11
	NAU3308	qLP-D2–1	8
	JESPR204	qLP-18–1	11, 20^A^
	BNL1672	qLP-D9–1	14
FS	DC40182	qFS07.1, qFS-C7–1	13, 39
	CIR307	qFS-chr1–1, qFS-C15–1	21, 42
	JESPR153		21^A^
	TMB1618	qFS-C7–1	12
FL	BNL1395	qFL-16–1	11
	DC40182	qFL-C7–1	39
	NAU2980	qFL-C18–1	12
	BNL2572	qFL-C25–2	12
FM	JESPR274	qFM-A9–1	43

^A^ means linkage analysis.

**Table 6:** Association markers closed to that linked or associated with the same traits in previous studies.

Trait	Marker associated	Marker reported	Distance (cM)	QTL	References
LP	BNL3590	JESPR101	1.3		19^A^
	NAU3995	NAU1167	0.4	qLPA3–2	41
	NAU3308	NAU4024	3.0		19 ^A^
	BNL1395	BNL1694	3.3	qLP-08A-c16–2	15
NB	cgr6807	NAU6584	0.7		20 ^A^
FL	CIR307	NAU2985	5.0	qFL-10–1	40
FS	NAU3736	CIR307	3.5	qFS-chr1–1, qFS-C15–1	21, 42
FM	NAU3700	BNL3590	11.0		19 ^A^
	JESPR101	BNL3590	12.2		19 ^A^

^A^ means marker nearby was identified by association analysis.

## Discussion

### Genetic diversity and population structure

To maintain relatively high levels of polymorphism and to take advantage of association mapping, different ecotypes from China, including lines from cotton germplasm resources, historical varieties from abroad (the Uzbek, the US, Australia, Cuba and Sudan), mutants lines derived from radiation breeding programs, and some progenies of intra- and interspecies crosses were employed in this panel. The results revealed an average genetic diversity, PIC and locus number of 0.36, 0.30 and 2.63, respectively. These results were consistent with that obtained in previous studies reporting genetic diversity, PIC and locus richness values of 0.34, 0.28 and 2.26 [22] or 0.32, 0.27 and 2.86 [20], respectively. A low genetic diversity was not only found in Chinese Upland cotton collections but also in American Upland cotton collections [38] and other country’s collections [44].

Population structure is an important factor that typically leads to spurious associations. Although the genetic background of Upland cotton is narrow, recent studies have revealed the population structure in association panels for Upland cotton [20–22, 38, 44]. Of 241 collections, 127 came from the Yangzi River valley, and 76 came from the Yellow River valley. In the present study, 73.2% (93/127) of the germplasm resources, varieties and breeding lines from the Yangtze River valley were classified into the P1 sub-group. A total of 75.0% (57/76) of the germplasm resources, varieties and breeding lines from the Yellow River valley were classified into the P2 sub-group. The results revealed that the major differences in this panel came from the different ecotypes. However, 25.0% (19/76) of the collections from the Yellow River valley and 26.8% (34/127) of the collections from the Yangzi River valley were not arranged into corresponding subgroups. The fact indicates that there is still frequent gene exchange between different ecotype collections in China (S1 Table). These results were consistent with the results of previous studies [45] and recent reports [20–22]. Evanno et al. [46] conducted population structure analyses using three classic models: the island model, the hierarchical island model and the contact zone model, and K = 2 corresponds to the uppermost structural level in the contact zone model. In this study, population structure was similar with that of the contact zone model. The result was consistent with the fact that China is not a native cotton growing area. Most cotton varieties planted in China are derived from only a few germplasm resources (e.g., DPL, Stoneville, King, Uganda, Foster, and Trice) introduced from abroad [47].

### Linkage disequilibrium

A successful association analysis depends on knowing the precise LD status of a population. In the present study, 9.36% of the marker pairs showed significant LD values, while 18.90% of collinear and 8.90% of non-collinear marker pairs showed significant LD. Compared with previous studies in which 22% of locus pairs [16], 21.03% of linked locus pairs, or 18.18% of unlinked pairs showed significant LD [20], the ratio of LD was low and similar to the findings of Zhao [22]. Among the collinear marker pairs, 29.2% showed LD values (r^2^) greater than 0.2. For the non-collinear marker pairs, this ratio was 0.5% (S2 Table). Further examination of the LD data revealed that approximately 80.5% of moderate LD (0.2 < r^2^ < 0.4) and 91.5% of strong LD (r^2^ > 0.4) was caused by linkage. Our results also showed that approximately 43.6% of moderate LD (r^2^ > 0.1) was caused by other factors in this panel. LD resulting from non-collinear marker pairs has been previously described [16, 20, 22, 44]. Abdurakhmonov [16] provided several possible explanations for LD between non-collinear markers, including selection, co-selection of loci, population stratification, and relatedness, genetic drift or bottlenecks. These elements might also generate LD values leading to spurious marker-trait associations [48–50], indicating the necessity of seriously considering population structure (Q) and relatedness (K) when conducting population-based association mapping in cotton germplasm resources [16].

In the present study, the observed LD value (r^2^) rapidly decreased when the genetic distance was less than 10 cM. The speed of population LD decay was 8.58 or 5.76 cM for r^2^ > 0.1 or 0.2, respectively. The LD decay block was similar to that described in recent association analysis studies [20–22] but faster than that described in studies using landrace [16, 17] and SP panels [18]. We selected markers that were spaced approximately 10 cM apart from the frame linkage map [23, 24]. Because of the shortage of polymorphism markers, there were some gaps of more than 15 to 46.8 cM along the 26 chromosomes. Although more markers are needed to conduct genome-wide association analyses (GWAS) of complex traits, the size of the LD blocks would guarantee that the identified SSR markers would be sufficient for MAS in Upland cotton breeding programs because increasing the number of markers per chromosome does not necessarily result in a stronger response to selection, particularly at a shorter distance between markers, such as 10 cM for an F_2_ population of 500 individuals [51].

### QTLs obtained through association mapping

To avoid spurious associations, different methods have been developed to control population structure, such as structured association (SA) [48], genomic control (GC) [52], EIGENSTRAT [53], stepwise regression (SWR) [54] and mixed linear models (MLM) [55]. To generate more accurate correlations with less-inflated type I errors [55], the MLM (+K+Q) method was employed in the present study. Considering the history of Upland cotton cultivation and the relatively simple population structure in this panel, GLM (+K) was also employed in the present study, and the results derived from the GLM and MLM were compared. For all six traits, the GLM (*p* < 0.05) detected 216 associated markers, and 155 markers were detected in more than one environment. The MLM (*p* < 0.05) identified 195 associated markers, and 84 markers were detected in more than one environment. After the correction of population structure using Q-matrix information, approximately 50% of the markers were not repeatedly detected in the MLM compared with the GLM, suggesting that the population structure should be seriously considered in stratified populations [17]. However, comparing the results obtained from the GLM and MLM provides more information. In the present study, all of the associated markers detected through the MLM were associated with the same traits in the GLM analysis across two to four environments. Notably, for the same traits, we compared the map positions of the associated markers derived from the GLM and MLM analyses, and we found more than one associated markers from the GLM were close to (within one or two LD blocks) associated markers detected using the MLM. This observation provided more support for the validity of the MLM results [17].

Interestingly, out of the 46 associated markers detected, some nearby markers (map distance within 1 LD block) were associated with the same traits. For example, both of NAU3736 and CIR307 on D1 were associated with FS and NB, JESPR101 and NAU3700 on D3 were associated with FM (S3 Fig.). These nearby markers might associate with the same QTL allele with a high probability.

Comparing the results derived from different populations or using different analytical approaches for cotton QTL detection provides more information for interpreting the results of the present study. Among all 46 markers associated with yield and fiber quality traits, 14 markers associated with the same traits were identified in previous studies (Table 5). Thirteen markers were detected through linkage analysis, and three markers were detected via association analysis. When we employed the reference map as a bridge to compare the results of the present study with those from previous studies, the 9 associated markers identified were near the QTL-linked/associated markers controlling the same traits identified in other reports, at distances of less than 1–2 LD decay on the reference map (Table 6). Considering the different markers used in the prior studies and the precision of QTL detection, these nearby marker pairs should be linked to the same QTLs reported.

MLM analysis generates more accurate correlations with less-inflated type I errors. However, significant MLM-derived associations are subjected to multiple testing corrections. The results of correction for multiple testing could be misleading due to the unknown influence of *p*-value adjustment methods applied under the MLM approach [17]. Perhaps a modified statistical approach should be applied to adjust MLM *p*-values, though answering this question will require further studies [17]. In the present study, to maintain low false positive results, we employed four environmental datasets and four different significance tests (*p*-value, BFmin, FDR and permutation testing). Although most of the associated markers did not tolerate multiple testing for the FDR, the results of the present study obtained using the MLM method were supported by the BFmin, FDR and permutation testing from the GLM analysis as well as the findings of previous studies. These results exhibited a relatively high confidence level and can be considered for use in MAS programs.

To date, few SSR markers have been efficiently employed in MAS programs in cotton because the majority of available marker information was derived from populations resulting from bi-parental crosses with limited genetic backgrounds, covering only a few meiotic events since experimental hybridization [17]. Recent association mapping of Upland cotton collections confirmed the feasibility of applying association analysis to explore complex traits in Upland cotton collections and provided useful markers for marker-assisted breeding programs [18–22]. Similar to linkage mapping, association mapping using different materials harboring different genes and different markers can provide more information for marker-assisted breeding programs as well as insight into the genetic basis of interesting traits in Upland cotton. The results of the present study provided new useful markers for marker-assisted selection in cotton breeding programs and clues for the fine mapping of yield and fiber quality traits. These results will also enhance our current understanding of the genetic basis of Upland cotton yield and fiber quality traits at the whole-genome level.

## Supporting Information

S1 Fig Cluster analysis of 241 Upland cotton collections. SSR allele frequencies were calculated with TASSEL 3.0 software; Colored symbols represent the subgroups where the collections were arranged by STRUCTURE. Red indicates subgroup 1 and green indicates subgroup 2.
(DOC)

S2 Fig Distribution of LD among all major loci on the 26 chromosomes in the panel. Loci were sorted according to their map location on A1–D13. The r^2^ between marker pairs is shown in different colored blocks.
(DOC)

S3 Fig Distribution of markers used in the analysis and associated with traits on the reference map. Associated markers are shown red; * indicates linked or associated with the same yield components traits in previous reports; ^+^ indicates separated from markers linked or associated with the same traits in previous reports by a distance less than 1–2 LD decay on the reference map.
(DOC)

S1 Table Subgroup arrangement and geographical origins of the 241 collections used in association mapping. ^**a**^ 1 Elite varieties that have been popularly cultivated in China; 2 Germplasm resource lines with outstanding character of yield component or fiber quality; 3 Parent lines used in the breeding program; 4 Non-domestic historical varieties and germplasm resources lines.
(DOC)

S2 Table Frequency distribution of LD (r^2^) of marker pairs in the 241 Upland cotton collections (*p* < 0.05). (DOC)

S3 Table Phenotypic performance of yield and fiber quality traits across four environments. (DOC)

S4 Table Mean squares of the ANOVA for yield and fiber quality traits of 241 collections in four environments. *Significant at the *p* < 0.0001 level.
(DOC)

## References

[1] Shappley ZW, Jenkins JN, Zhu J, McCarty JC (1998) Quantitative trait loci associated with agronomic and fiber traits of Upland cotton. J Cotton Science, 2(4):153–163

[2] Zhang ZS, Xiao YH, Luo M, Li XB, Luo XY, et al (2005) Construction of a genetic linkage map and QTL analysis of fiber-related traits in Upland cotton (*Gossypium hirsutum* L.). Euphyt 144:91–99

[3] Zhang ZS, Rong JK, Waghmare VN, Chee PW, May OL, et al (2011) QTL alleles for improved fiber Quality from a wild Hawaii cotton, *Gossypium tomentosum* . Theor Appl Genet 123(7):1075–1088 10.1007/s00122-011-1649-x 21735234

[4] Shen XL, Guo WZ, Zhu XF, Yuan YL, Yu JZ, et al (2005) Molecular mapping of QTLs for fiber qualities in three diverse lines in Upland cotton using SSR markers. Mol Breed 15:169–181

[5] Shen XL, Guo WZ, Lu QX, Zhu XF, Yuan YL, et al (2007) Genetic mapping of quantitative trait loci for fiber quality and yield trait by RIL approach in Upland cotton. Euphytica 155:371–380

[6] Wang BH, Guo WZ, Zhu XF, Wu YT, Huang NT, et al (2006) QTL mapping of fiber quality in an elite hybrid derived-RIL population of Upland cotton. Euphyt 152:367–378 16436425

[7] Zhang JH, Wang SF, Shi YZ, Zhang GY, Liu AY, et al (2008) Molecular Marker and QTL of Yield-related Traits in Transgenic Insect-resistant Cotton Varieties. Cotton Sci Sini 20 (3):179–185

[8] Qin HD, Guo WZ, Zhang Y-M, Zhang TZ (2008) QTL mapping of yield and fiber traits based on a four-way cross population in *Gossypium hirsutum* L. Theor Appl Genet 117:883–894 10.1007/s00122-008-0828-x 18604518

[9] Li CQ, Guo WZ, Ma XL, Zhang TZ (2008) Tagging and Mapping of QTL for Yield and its Components in Upland Cotton (*Gossypium hirsutum* L.) Population with Varied Lint Percentage. Cotton Sci Sini 20(3):163–169

[10] Wu JX, Gutierrez OA, Jenkins JN, McCarty JC, Zhu J (2009) Quantitative analysis and QTL mapping for agronomic and fiber traits in an RI population of Upland cotton. Euphyt 165(2):231–245

[11] Wang FR, Gong YC, Zhang CY, Liu GD, Wang LM, et al (2011) Genetic effects of introgression genomic components from Sea Island cotton (*Gossypium barbadense* L.) on fiber related traits in Upland cotton (*G*. *hirsutum* L.). Euphyt 181:41–53

[12] Sun FD, Zhang JH, Wang SF, Gong WK, Shi YZ, et al (2012) QTL mapping for fiber quality traits across multiple generations and environments in Upland cotton. Mol Breed. 30(1):569–582

[13] Zhang K, Zhang J, Ma J, Tang SY, Liu DJ, et al (2012) Genetic mapping and quantitative trait locus analysis of fiber quality traits using a three-parent composite population in Upland cotton (*Gossypium hirsutum* L.). Mol Breed 29(2):335–348 10.1089/neu.2011.1862 21806472

[14] Liu RZ, Wang BH, Guo WZ, Qin YS, Wang LG, et al (2012) Quantitative trait loci mapping for yield and its components by using two immortalized populations of a heterotic hybrid in *Gossypium hirsutum* L. Mol Breed 29:297–311

[15] Yu JW, Zhang K, Li SY, Yu SX, Zhai HH, et al (2013) Mapping quantitative trait loci for lint yield and fiber quality across environments in a *Gossypium hirsutum* × *Gossypium barbadense* backcross inbred line population. Theor Appl Genet 126:275–287 10.1007/s00122-012-1980-x 23064252

[16] Abdurakhmonov IY, Kohel R, Yu JZ, Pepper AE, Abdullaev AA, et al (2008) Molecular diversity and association mapping of fiber quality traits in exotic *G*. *hirsutum* L. germgermplasm. Genomics 92(6):478–87 10.1016/j.ygeno.2008.07.013 18801424

[17] Abdurakhmonov IY, Saha S, Jenkins JN, Buriev ZT, Shermatov SE, et al (2009) A Linkage disequilibrium based association mapping of fiber quality traits in *G*. *hirsutum* L. Variety germplasm. Genetica 136 (3):401–417 10.1007/s10709-008-9337-8 19067183

[18] Zeng LH, Meredith WR Jr, Gutiérrez OA, Boykin DL (2009) Identification of associations between SSR markers and fiber traits in an exotic germplasm derived from multiple crosses among *Gossypium* tetraploid species. Theor Appl Genet 119(1):93–103 10.1007/s00122-009-1020-7 19360391

[19] Zhang TZ, Qian N, Zhu XF, Chen H, Wang S, et al (2013) Variations and Transmission of QTL Alleles for Yield and Fiber Qualities in Upland Cotton Cultivars Developed in China. PLOS ONE 8(2): e57220 10.1371/journal.pone.0057220 23468939 PMC3584144

[20] Mei HX, Zhu XF, Zhang TZ (2013) Favorable QTL Alleles for Yield and Its Components Identified by Association Mapping in Chinese Upland Cotton Cultivars. PLOS ONE 8 (12):e82193 10.1371/journal.pone.0082193 24386089 PMC3873261

[21] Cai CP, Ye WX, Zhang TZ, Guo WZ (2014) Association analysis of fiber quality traits and exploration of elite alleles in Upland cotton cultivars/accessions (*Gossypium hirsutum* L.). Jour Integrat Plant Biol 56(1):51–62 10.1111/jipb.12124 24428209

[22] Zhao YL, Wang HM, Wei CW, Li YH (2014) Genetic Structure, Linkage Disequilibrium and Association Mapping of Verticillium Wilt Resistance in Elite Cotton (*Gossypium hirsutum* L.) Germplasm Population. PLOS ONE 9 (1): e86308 10.1371/journal.pone.0086308 24466016 PMC3900507

[23] Guo WZ, Cai CP, Wang CB, Han ZG, Song XL, et al (2007) A microsatellite-based, gene-rich linkage map reveals genome structure, function, and evolution in *Gossypium* . Genetics 176:527–541 17409069 10.1534/genetics.107.070375 PMC1893075

[24] Liang Z, Lv YD, Cai CP, Tong XC, Chen XD, et al (2012) Toward allotetraploid cotton genome assembly: integration of a high-density molecular genetic linkage map with DNA sequence information. BMC Genomics 13:539 10.1186/1471-2164-13-539 23046547 PMC3557173

[25] Paterson AH, Brubaker CL, Wendel JF (1993) A rapid method for extraction of cotton (*Gossypium spp*) genomic DNA suitable for RFLP or PCR analysis. Plan Mol Biol Rep 11:122–127

[26] Fountain J, Qin H, Chen C, Dang P, Wang ML, et al (2011) A note on development of a low-cost and high-throughput SSR-based genotyping method in peanut (*Arachis hypogaea* L.). Peanut Sci 38:1–7

[27] Bradbury PJ, Zhang Z, Kroon DE, Casstevens TM, Ramdoss Y, et al (2007) TASSEL: software for association mapping of complex traits in diverse samples. Bioinformatics 23(19):2633–26355 17586829 10.1093/bioinformatics/btm308

[28] Mohlke KL, Lange EM, Valle TT, Ghosh S, Magnuson VL, et al (2001) Linkage disequilibrium between microsatellite markers extends beyond 1 cM on chromosome 20 in Finns. Genome Res 11:1221–1226 11435404 10.1101/gr.173201 PMC311096

[29] McRae AF, McEwan JC, Dodds KG, Wilson T, Crawford AM, et al (2002) Linkage disequilibrium in domestic sheep. Genetics 160:1113–1122 11901127 10.1093/genetics/160.3.1113 PMC1462013

[30] Witt SR, Buckler ES (2003) Using natural allelic diversity to evaluate gene function. Methods Mol Biol 236:123–139 14501062 10.1385/1-59259-413-1:123

[31] Pritchard JK, Stephens M, Donnelly P (2000) Inference of population structure using multilocus genotype data. Genetics 155:945–959 10835412 10.1093/genetics/155.2.945 PMC1461096

[32] Falush D, Stephens M, Pritchard JK (2003) Inference of population structure: Extensions to linked loci and correlated allele frequencies. Genetics 164:1567–1587 12930761 10.1093/genetics/164.4.1567 PMC1462648

[33] Falush D, Stephens M, Pritchard JK (2007) Inference of population structure using multilocus genotype data: dominant markers and null alleles. Mol Ecol Notes 7:574–587 18784791 10.1111/j.1471-8286.2007.01758.x PMC1974779

[34] Storey JD, Tibshirani R (2003) Statistical significance for genomewide studies. PNAS 100(16):9440–9445 12883005 10.1073/pnas.1530509100 PMC170937

[35] Goodman SN (2001) Of P-values and Bayes: a modest proposal. Epidemiology 12:295–297. 11337600 10.1097/00001648-200105000-00006

[36] Katki HA (2008) Invited commentary: evidence-based evaluation of P values and Bayes factors. Am J Epidemiol 268:384–388

[37] Mezmouk S, Dubreuil P, Bosio M, Décousset L, Charcosset A, et al (2011) Effect of population structure corrections on the results of association mapping tests in complex maize diversity panels. Theor Appl Genet 122(6):1149–1160 10.1007/s00122-010-1519-y 21221527 PMC3057001

[38] Tyagi P, Gore MA, Bowman DT, Campbell BT, Udall JA, et al (2014) Genetic diversity and population structure in the US Upland cotton (*Gossypium hirsutum* L.). Theor Appl Genet 127 (2):283–295 10.1007/s00122-013-2217-3 24170350

[39] Wang FR, Xu ZZ, Sun R, Gong YC, Liu GD, et al (2013) Genetic dissection of the introgressive genomic components from *Gossypium barbadense* L. that contribute to improved fiber quality in *Gossypium hirsutum* L. Mol Breed 32(3):547–562

[40] Wang L, Liu F, Li SH, Wang CY, Zhang XD, et al (2012) QTL mapping for fiber quality properties in Lumianyan15. Cotton Sci Sini 24(2): 97–105

[41] Qin YS, Liu RZ, Mei HX, Zhang TZ, Guo WZ (2009) QTL Mapping for Yield Traits in Upland Cotton (*Gossypium hirsutum* L.). Acta Agronomica Sinica 35(10): 1812–1821

[42] Liang QZ, Hu C, Hua H, Li ZH, Hua JP (2013) Construction of a linkage map and QTL mapping for fiber quality traits in upland cotton (*Gossypium hirsutum* L.). Chin Sci Bull 58:3233–3243

[43] Wang P, Zhu YJ, Song XL, Cao ZB, Ding YZ, et al (2012) Inheritance of long staple fiber quality traits of *Gossypium barbadense* in *G*.*hirsutum* background using CSILs. Theor Appl Genet 124(8):1415–1428 10.1007/s00122-012-1797-7 22297564

[44] Fang DD, Hinze LL, Percy RG, Li P, Deng D, et al (2013) A microsatellite-based genome-wide analysis of genetic diversity and linkage disequilibrium in Upland cotton (*Gossypium hirsutum* L.) cultivars from major cotton-growing countries. Euphyt 191(3):391–401

[45] He DH, Xing HY, Li TT, Tang Y, Zeng Z (2010) Genetic diversity of 92 cotton accessions evaluated with SSR markers. Acta Bot Borea Occident Sin 30(8):1557–156

[46] Evanno G, Regnaut S, Goudet J (2005) Detecting the number of clusters of individuals using the software STRUCTURE: a simulation study. Mol Ecol 14:2611–2620 15969739 10.1111/j.1365-294X.2005.02553.x

[47] Liu S, Cantrell RG, Mcarty JC J, Stewart JM (2000) Simple sequence repeatbased assessment of genetic diversity in cotton race stock accessions. Crop Sci 40:1459–1469

[48] Pritchard JK, Stephens M, Rosenberg NA, Donnelly P (2000) Association mapping in structured populations. Am J Hum Genet 67:170–181 10827107 10.1086/302959 PMC1287075

[49] Stich B, Melchinger AE, Frisch M, Maurer HP, Heckenberger M, et al (2005) Linkage disequilibrium in European elite maize germplasm investigated with SSRs. Theor Appl Genet 111:723–730 15997389 10.1007/s00122-005-2057-x

[50] Stich B, Maurer HP, Melchinger AE, Frisch M, Heckenberger M, et al (2006) Comparison of linkage disequilibrium in elite European maize inbred lines using AFLP and SSR markers. Mol Breeding 17(3):217–226

[51] Gimeifarb A, Lande R (1995) Marker-assisted selection and marker-QTL associations in hybrid populations. Theor Appl Genet 91:522–528 10.1007/BF00222983 24169845

[52] Devlin B, Roeder K (1999) Genomic control for association studies. Biometrics 55:997–1004 11315092 10.1111/j.0006-341x.1999.00997.x

[53] Price AL, Patterson NJ, Plenge RM, Weinblatt ME, Shadick NA, et al (2006) Principal components analysis corrects for stratification in genome-wide association studies. Nat Genet 38:904–909 16862161 10.1038/ng1847

[54] Setakis E, Stirnadel H, Balding DJ (2006) Logistic regression protects against population structure in genetic association studies. Genome Res 16:290–296 16354752 10.1101/gr.4346306 PMC1361725

[55] Yu JM, Pressoir G, Briggs WH, Bi IV, Yamasaki M, et al (2006) A unified mixed-model method for association mapping that accounts for multiple levels of relatedness. Nat Genet 38:203–208 16380716 10.1038/ng1702

