# Supplementary material for: Identification of Associated SSR Markers for Yield Component and Fiber Quality Traits Based on Frame Map and Upland Cotton Collections
Source: PLoS One. 2015 Jan 30;10(1):e0118073. doi: 10.1371/journal.pone.0118073 (PMC4311988; doi:10.1371/journal.pone.0118073)
Supplement: S1 Fig — SSR allele frequencies were calculated with TASSEL 3.0 software; Colored symbols represent the subgroups where the collections were arranged by STRUCTURE. Red indicates subgroup 1 and green indicates subgroup 2. (DOC) [file pone.0118073.s001.doc]

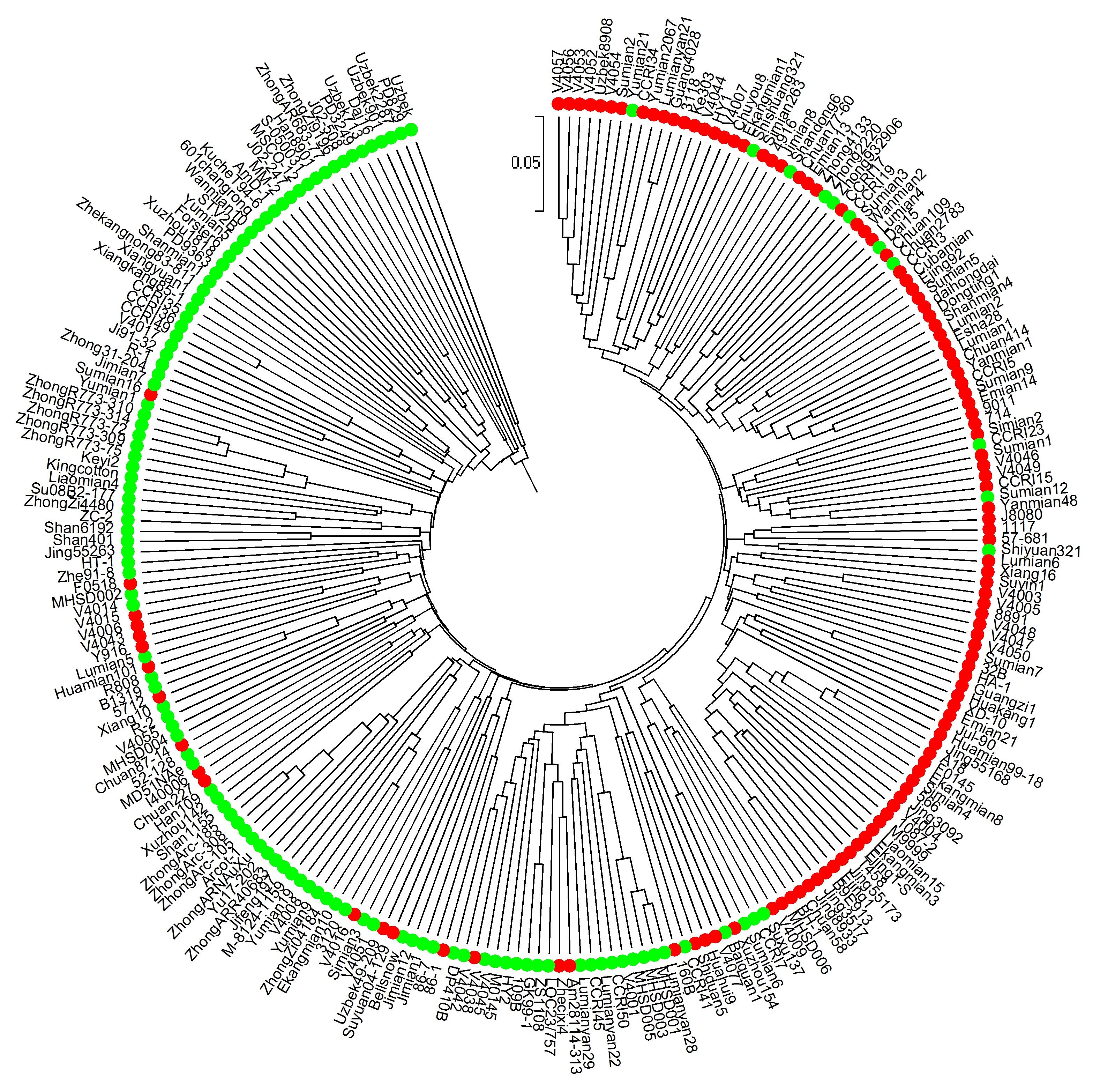


**Figure S1.** **Cluster analysis of 241 Upland cotton collections.** SSR allele frequencies were calculated with TASSEL 3.0 software; Colored symbols represent the subgroups where the collections were arranged by STRUCTURE. Red indicates subgroup 1 and green indicates subgroup 2.
