# Supplementary material for: Identification of Associated SSR Markers for Yield Component and Fiber Quality Traits Based on Frame Map and Upland Cotton Collections
Source: PLoS One. 2015 Jan 30;10(1):e0118073. doi: 10.1371/journal.pone.0118073 (PMC4311988; doi:10.1371/journal.pone.0118073)
Supplement: S2 Fig — Loci were sorted according to their map location on A1–D13. The r2 between marker pairs is shown in different colored blocks. (DOC) [file pone.0118073.s002.doc]

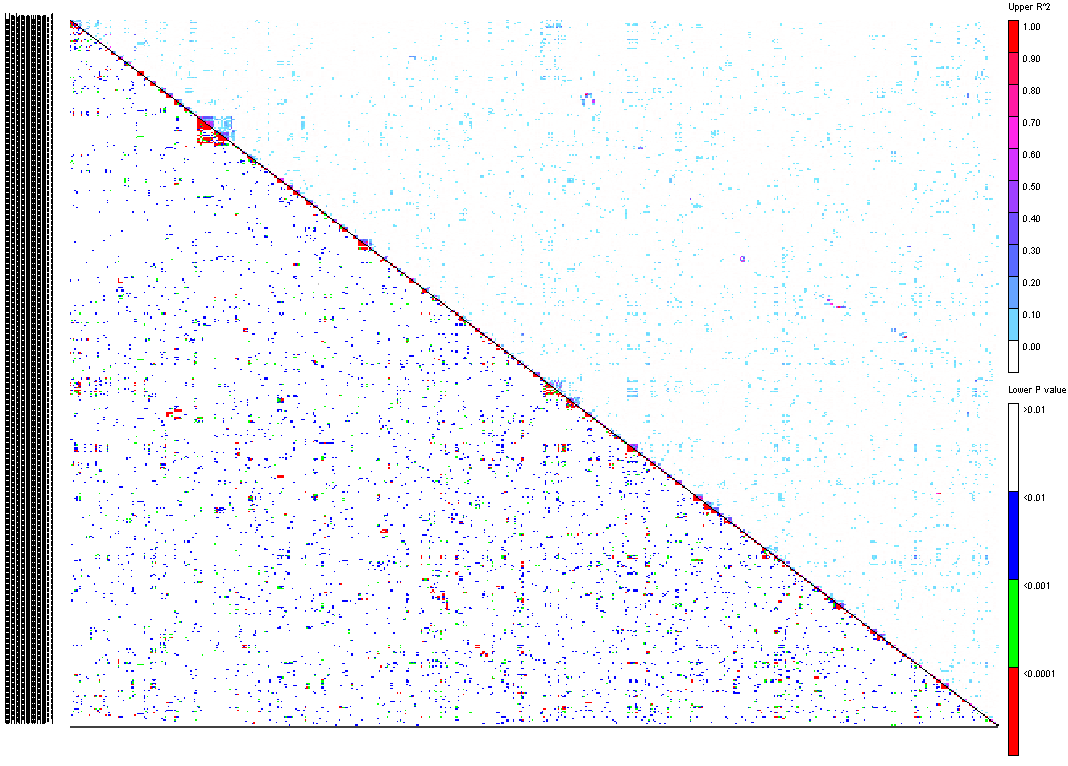


**Figure S2.** **Distribution of LD among all major loci on the 26 chromosomes in the panel.** Loci were sorted according to their map location on A1-D13. The r^2^ between marker pairs is shown in different colored blocks.
