# Supplementary material for: Identification of Associated SSR Markers for Yield Component and Fiber Quality Traits Based on Frame Map and Upland Cotton Collections
Source: PLoS One. 2015 Jan 30;10(1):e0118073. doi: 10.1371/journal.pone.0118073 (PMC4311988; doi:10.1371/journal.pone.0118073)
Supplement: S3 Fig — Associated markers are shown red; * indicates linked or associated with the same yield components traits in previous reports; + indicates separated from markers linked or associated with the same traits in previous reports by a distance less than 1–2 LD decay on the reference map. (DOC) [file pone.0118073.s003.doc]

**Figure S3. Distribution of markers used in the analysis and associated with traits on the reference map.** Associated markers are shown red; ***** indicates linked or associated with the same yield components traits in previous reports**; ^+^** indicates separated from markers linked or associated with the same traits in previous reports by a distance less than 1-2 LD decay on the reference map.
