# Supplementary material for: Identification of Associated SSR Markers for Yield Component and Fiber Quality Traits Based on Frame Map and Upland Cotton Collections
Source: PLoS One. 2015 Jan 30;10(1):e0118073. doi: 10.1371/journal.pone.0118073 (PMC4311988; doi:10.1371/journal.pone.0118073)
Supplement: S1 Table — a 1 Elite varieties that have been popularly cultivated in China; 2 Germplasm resource lines with outstanding character of yield component or fiber quality; 3 Parent lines used in the breeding program; 4 Non-domestic historical varieties and germplasm resources lines. (DOC) [file pone.0118073.s004.doc]

**Table S1.** Subgroup arrangement and geographical origins of the 241 collections used in association mapping

| **Subgroups** | **Collections Name** | **Q1** | **Q2** | **Type**^a^ | **Geographical Origin** |
| --- | --- | --- | --- | --- | --- |
| 1 | Ejing1-S | 0.0164 | 0.9836 | 3 | the Yangzi River valley, China |
| 1 | Jing8933 | 0.0173 | 0.9827 | 1 | the Yangzi River valley, China |
| 1 | Ejing1 | 0.0213 | 0.9787 | 1 | the Yangzi River valley, China |
| 1 | M9999 | 0.0226 | 0.9774 | 3 | the Yangzi River valley, China |
| 1 | Jing3517 | 0.0245 | 0.9755 | 1 | the Yangzi River valley, China |
| 1 | 8891 | 0.025 | 0.975 | 3 | the Yangzi River valley, China |
| 1 | 1087-2 | 0.0259 | 0.9741 | 2 | the Yangzi River valley, China |
| 1 | Jing55168 | 0.029 | 0.971 | 3 | the Yangzi River valley, China |
| 1 | Ekangmian3 | 0.0299 | 0.9701 | 1 | the Yangzi River valley, China |
| 1 | A18 | 0.0304 | 0.9696 | 3 | the Yangzi River valley, China |
| 1 | Jing55173 | 0.0305 | 0.9695 | 1 | the Yangzi River valley, China |
| 1 | daihongdai | 0.0314 | 0.9686 | 1 | the Yangzi River valley, China |
| 1 | V4004 | 0.036 | 0.964 | 3 | the Yangzi River valley, China |
| 1 | Ekangmian8 | 0.0417 | 0.9583 | 1 | the Yangzi River valley, China |
| 1 | T458 | 0.0469 | 0.9531 | 2 | the Yangzi River valley, China |
| 1 | Jing3092 | 0.0495 | 0.9505 | 3 | the Yangzi River valley, China |
| 1 | Ekangmian1 | 0.0584 | 0.9416 | 1 | the Yangzi River valley, China |
| 1 | F0145 | 0.0596 | 0.9404 | 3 | the Yangzi River valley, China |
| 1 | Huakang1 | 0.0701 | 0.9299 | 1 | the Yangzi River valley, China |
| 1 | Dongting1 | 0.0716 | 0.9284 | 1 | the Yangzi River valley, China |
| 1 | B9E913 | 0.0783 | 0.9217 | 2 | the Yangzi River valley, China |
| 1 | Liaomian15 | 0.0972 | 0.9028 | 1 | Northern Specific early maturation, China |
| 1 | V4007 | 0.1036 | 0.8964 | 3 | the Yangzi River valley, China |
| 1 | Guangzi1 | 0.1144 | 0.8856 | 3 | the Yangzi River valley, China |
| 1 | Huamian99-18 | 0.1392 | 0.8608 | 3 | the Yangzi River valley, China |
| 1 | XD-10 | 0.1434 | 0.8566 | 3 | the Yangzi River valley, China |
| 1 | Simian4 | 0.1487 | 0.8513 | 1 | the Yangzi River valley, China |
| 1 | Emian21 | 0.153 | 0.847 | 1 | the Yangzi River valley, China |
| 1 | UZBEK8908 | 0.1654 | 0.8346 | 4 | the Uzbek |
| 1 | Wanmian2 | 0.1686 | 0.8314 | 1 | the Yangzi River valley, China |
| 1 | V4048 | 0.1719 | 0.8281 | 3 | the Yangzi River valley, China |
| 1 | V4053 | 0.1808 | 0.8192 | 3 | the Yangzi River valley, China |
| 1 | CCRI5 | 0.1921 | 0.8079 | 1 | the Yellow River valley, China |
| 1 | Simian263 | 0.2019 | 0.7981 | 3 | the Yangzi River valley, China |
| 1 | Emian13 | 0.2045 | 0.7955 | 1 | the Yangzi River valley, China |
| 1 | Ke303 | 0.205 | 0.795 | 1 | the Yangzi River valley, China |
| 1 | Jul-90 | 0.2077 | 0.7923 | 3 | the Yangzi River valley, China |
| 1 | V4050 | 0.2128 | 0.7872 | 3 | the Yangzi River valley, China |
| 1 | Sumian7 | 0.2135 | 0.7865 | 1 | the Yangzi River valley, China |
| 1 | Ejing92 | 0.2257 | 0.7743 | 1 | the Yangzi River valley, China |
| 1 | Chuan77-60 | 0.2295 | 0.7705 | 3 | the Yangzi River valley, China |
| 1 | CCRI3 | 0.2304 | 0.7696 | 1 | the Yellow River valley, China |
| 1 | Esha28 | 0.2311 | 0.7689 | 1 | the Yangzi River valley, China |
| 1 | Lumian1 | 0.2314 | 0.7686 | 1 | the Yellow River valley, China |
| 1 | V4056 | 0.2332 | 0.7668 | 3 | the Yangzi River valley, China |
| 1 | Chuyou8 | 0.238 | 0.762 | 3 | the Yangzi River valley, China |
| 1 | V4044 | 0.2396 | 0.7604 | 3 | the Yangzi River valley, China |
| 1 | 9011 | 0.2465 | 0.7535 | 3 | the Yangzi River valley, China |
| 1 | Huahui9 | 0.2474 | 0.7526 | 1 | the Yangzi River valley, China |
| 1 | 714 | 0.2496 | 0.7504 | 3 | the Yangzi River valley, China |
| 1 | Sumian5 | 0.2574 | 0.7426 | 1 | the Yangzi River valley, China |
| 1 | V4057 | 0.2576 | 0.7424 | 3 | the Yangzi River valley, China |
| 1 | Yanmian1 | 0.2606 | 0.7394 | 1 | the Yangzi River valley, China |
| 1 | Emian14 | 0.2621 | 0.7379 | 1 | the Yangzi River valley, China |
| 1 | Simian2 | 0.2708 | 0.7292 | 1 | the Yangzi River valley, China |
| 1 | Sumian1 | 0.2753 | 0.7247 | 1 | the Yangzi River valley, China |
| 1 | Chuan414 | 0.2808 | 0.7192 | 1 | the Yangzi River valley, China |
| 1 | HY1 | 0.2812 | 0.7188 | 3 | the Yangzi River valley, China |
| 1 | Shiduan5 | 0.2885 | 0.7115 | 1 | the Yellow River valley, China |
| 1 | Yanmian48 | 0.2925 | 0.7075 | 1 | the Yangzi River valley, China |
| 1 | Suyin1 | 0.2927 | 0.7073 | 1 | the Yangzi River valley, China |
| 1 | V4038 | 0.3026 | 0.6974 | 3 | the Yangzi River valley, China |
| 1 | V4043 | 0.3064 | 0.6936 | 3 | the Yangzi River valley, China |
| 1 | 566 | 0.3073 | 0.6927 | 3 | the Yellow River valley, China |
| 1 | 4916 | 0.3075 | 0.6925 | 3 | the Yangzi River valley, China |
| 1 | V4003 | 0.3082 | 0.6918 | 3 | the Yangzi River valley, China |
| 1 | J8080 | 0.3143 | 0.6857 | 2 | the Yangzi River valley, China |
| 1 | V4077 | 0.3224 | 0.6776 | 3 | the Yangzi River valley, China |
| 1 | V4049 | 0.3224 | 0.6776 | 3 | the Yangzi River valley, China |
| 1 | 86-1 | 0.3242 | 0.6758 | 1 | the Yellow River valley, China |
| 1 | Zhecixi4 | 0.3337 | 0.6663 | 1 | the Yangzi River valley, China |
| 1 | CCRI15 | 0.338 | 0.662 | 1 | the Yellow River valley, China |
| 1 | V4046 | 0.3388 | 0.6612 | 3 | the Yangzi River valley, China |
| 1 | Xiang16 | 0.3428 | 0.6572 | 1 | the Yangzi River valley, China |
| 1 | V4047 | 0.3521 | 0.6479 | 3 | the Yangzi River valley, China |
| 1 | Shanmian4 | 0.353 | 0.647 | 1 | the Yellow River valley, China |
| 1 | Lumian4 | 0.3533 | 0.6467 | 1 | the Yellow River valley, China |
| 1 | 1117 | 0.3543 | 0.6457 | 3 | the Yangzi River valley, China |
| 1 | BH-1 | 0.3567 | 0.6433 | 2 | the Yangzi River valley, China |
| 1 | 32B | 0.36 | 0.64 | 4 | U.S.A. |
| 1 | 3118 | 0.3634 | 0.6366 | 3 | the Yangzi River valley, China |
| 1 | Sumian2 | 0.3674 | 0.6326 | 1 | the Yangzi River valley, China |
| 1 | Yumian2067 | 0.3681 | 0.6319 | 3 | the Yellow River valley, China |
| 1 | Cubamian | 0.3744 | 0.6256 | 4 | Cuba |
| 1 | V4009 | 0.3848 | 0.6152 | 3 | the Yangzi River valley, China |
| 1 | 160B | 0.3856 | 0.6144 | 4 | U.S.A. |
| 1 | Chuan87-14 | 0.3991 | 0.6009 | 3 | the Yangzi River valley, China |
| 1 | V4052 | 0.4003 | 0.5997 | 3 | the Yangzi River valley, China |
| 1 | I40006 | 0.4018 | 0.5982 | 3 | the Yangzi River valley, China |
| 1 | CCRI17 | 0.4053 | 0.5947 | 1 | the Yellow River valley, China |
| 1 | MHSD006 | 0.4115 | 0.5885 | 4 | Sudan |
| 1 | Zhong4133 | 0.412 | 0.588 | 3 | the Yellow River valley, China |
| 1 | Chuan109 | 0.4141 | 0.5859 | 3 | the Yangzi River valley, China |
| 1 | Sumian3 | 0.4208 | 0.5792 | 1 | the Yangzi River valley, China |
| 1 | V4005 | 0.4218 | 0.5782 | 3 | the Yangzi River valley, China |
| 1 | HA-1 | 0.4268 | 0.5732 | 2 | the Yangzi River valley, China |
| 1 | Chuan58 | 0.4269 | 0.5731 | 3 | the Yangzi River valley, China |
| 1 | 5712 | 0.4274 | 0.5726 | 3 | the Yangzi River valley, China |
| 1 | Shishuang321 | 0.4317 | 0.5683 | 1 | the Yellow River valley, China |
| 1 | Am28114-313 | 0.4326 | 0.5674 | 4 | U.S.A. |
| 1 | Lumianyan21 | 0.4334 | 0.5666 | 1 | the Yellow River valley, China |
| 1 | Huamian101 | 0.4415 | 0.5585 | 1 | the Yangzi River valley, China |
| 1 | V4015 | 0.4462 | 0.5538 | 3 | the Yangzi River valley, China |
| 1 | V4016 | 0.4483 | 0.5517 | 3 | the Yangzi River valley, China |
| 1 | F0518 | 0.449 | 0.551 | 3 | the Yangzi River valley, China |
| 1 | Guang4028 | 0.4497 | 0.5503 | 3 | the Yangzi River valley, China |
| 1 | CCRI34 | 0.4498 | 0.5502 | 1 | the Yellow River valley, China |
| 1 | UZBEK49-209 | 0.4511 | 0.5489 | 4 | the Uzbek |
| 1 | Y916 | 0.4606 | 0.5394 | 3 | the Yangzi River valley, China |
| 1 | V4006 | 0.4668 | 0.5332 | 3 | the Yangzi River valley, China |
| 1 | Sumian9 | 0.4671 | 0.5329 | 1 | the Yangzi River valley, China |
| 1 | Lumian2 | 0.4738 | 0.5262 | 1 | the Yellow River valley, China |
| 1 | Sumian16 | 0.4779 | 0.5221 | 1 | the Yangzi River valley, China |
| 1 | Xuzhou154 | 0.4841 | 0.5159 | 3 | the Yellow River valley, China |
| 1 | V4054 | 0.487 | 0.513 | 3 | the Yangzi River valley, China |
| 1 | 57-681 | 0.4914 | 0.5086 | 3 | the Yangzi River valley, China |
| 1 | Suyuan04-129 | 0.4957 | 0.5043 | 2 | the Yangzi River valley, China |
| 1 | Lumian6 | 0.4957 | 0.5043 | 1 | the Yellow River valley, China |
| 1 | Chuan227 | 0.497 | 0.503 | 3 | the Yangzi River valley, China |
| 1 | Jimian8 | 0.4987 | 0.5013 | 1 | the Yellow River valley, China |
| 2 | Shiyuan321 | 0.502 | 0.498 | 1 | the Yellow River valley, China |
| 2 | Xuzhou142 | 0.5041 | 0.4959 | 1 | the Yellow River valley, China |
| 2 | MHSD005 | 0.5043 | 0.4957 | 4 | Sudan |
| 2 | HT-1 | 0.5069 | 0.4931 | 2 | the Yangzi River valley, China |
| 2 | Lumian5 | 0.5076 | 0.4924 | 1 | the Yellow River valley, China |
| 2 | Dai15 | 0.5098 | 0.4902 | 1 | U.S.A. |
| 2 | V4045 | 0.5124 | 0.4876 | 3 | the Yangzi River valley, China |
| 2 | Yumian21 | 0.5192 | 0.4808 | 1 | the Yellow River valley, China |
| 2 | CCRI7 | 0.5247 | 0.4753 | 1 | the Yellow River valley, China |
| 2 | MHSD001 | 0.5248 | 0.4752 | 4 | Sudan |
| 2 | Xiang10 | 0.5254 | 0.4746 | 1 | the Yangzi River valley, China |
| 2 | V4008 | 0.5268 | 0.4732 | 3 | the Yangzi River valley, China |
| 2 | Suxu137 | 0.5287 | 0.4713 | 1 | the Yangzi River valley, China |
| 2 | CCRI19 | 0.5351 | 0.4649 | 1 | the Yellow River valley, China |
| 2 | Zhe91-8 | 0.5414 | 0.4586 | 3 | the Yangzi River valley, China |
| 2 | Jing55263 | 0.5485 | 0.4515 | 3 | the Yangzi River valley, China |
| 2 | ZhongARR40683 | 0.5509 | 0.4491 | 2 | the Yellow River valley, China |
| 2 | Chuan2783 | 0.5528 | 0.4472 | 3 | the Yangzi River valley, China |
| 2 | MHSD003 | 0.5541 | 0.4459 | 4 | Sudan |
| 2 | Xiangyuan1 | 0.5556 | 0.4444 | 1 | the Yangzi River valley, China |
| 2 | Sumian12 | 0.5583 | 0.4417 | 1 | the Yangzi River valley, China |
| 2 | V4051 | 0.559 | 0.441 | 3 | the Yangzi River valley, China |
| 2 | Jimian12 | 0.5603 | 0.4397 | 1 | the Yellow River valley, China |
| 2 | MHSD004 | 0.5637 | 0.4363 | 4 | Sudan |
| 2 | Shan1155 | 0.5695 | 0.4305 | 3 | the Yellow River valley, China |
| 2 | 86-7 | 0.5717 | 0.4283 | 1 | the Yellow River valley, China |
| 2 | Shandong6 | 0.5726 | 0.4274 | 1 | the Yellow River valley, China |
| 2 | CCRI49 | 0.5736 | 0.4264 | 1 | the Yellow River valley, China |
| 2 | Jimian1 | 0.5756 | 0.4244 | 1 | the Yellow River valley, China |
| 2 | ZS1108 | 0.5804 | 0.4196 | 3 | the Yangzi River valley, China |
| 2 | Ekangmian10 | 0.5818 | 0.4182 | 1 | the Yangzi River valley, China |
| 2 | R-2 | 0.5955 | 0.4045 | 2 | the Yangzi River valley, China |
| 2 | HY2 | 0.6009 | 0.3991 | 3 | the Yangzi River valley, China |
| 2 | GK99-1 | 0.6063 | 0.3937 | 1 | the Yangzi River valley, China |
| 2 | Simian3 | 0.6064 | 0.3936 | 1 | the Yangzi River valley, China |
| 2 | Sumian6 | 0.6069 | 0.3931 | 1 | the Yangzi River valley, China |
| 2 | MHSD002 | 0.6137 | 0.3863 | 4 | Sudan |
| 2 | MD51NAe | 0.6141 | 0.3859 | 4 | U.S.A. |
| 2 | Lumianyan28 | 0.6202 | 0.3798 | 1 | the Yellow River valley, China |
| 2 | M0145 | 0.6253 | 0.3747 | 3 | the Yangzi River valley, China |
| 2 | Lumianyan22 | 0.6308 | 0.3692 | 1 | the Yellow River valley, China |
| 2 | CCRI41 | 0.6349 | 0.3651 | 1 | the Yellow River valley, China |
| 2 | V4001 | 0.654 | 0.346 | 3 | the Yangzi River valley, China |
| 2 | 109B | 0.661 | 0.339 | 4 | U.S.A. |
| 2 | Bellsnow | 0.6641 | 0.3359 | 4 | U.S.A. |
| 2 | V4055 | 0.6647 | 0.3353 | 3 | the Yangzi River valley, China |
| 2 | Lumianyan29 | 0.6689 | 0.3311 | 1 | the Yellow River valley, China |
| 2 | Han109 | 0.6815 | 0.3185 | 1 | the Yellow River valley, China |
| 2 | Baiquan1 | 0.6839 | 0.3161 | 1 | the Yellow River valley, China |
| 2 | ZC-2 | 0.6854 | 0.3146 | 3 | the Yangzi River valley, China |
| 2 | CCRI50 | 0.6861 | 0.3139 | 1 | the Yellow River valley, China |
| 2 | Zhong2220 | 0.6863 | 0.3137 | 3 | the Yellow River valley, China |
| 2 | Wanmian10 | 0.692 | 0.308 | 1 | the Yangzi River valley, China |
| 2 | V4042 | 0.6939 | 0.3061 | 3 | the Yangzi River valley, China |
| 2 | CCRI45 | 0.6953 | 0.3047 | 1 | the Yellow River valley, China |
| 2 | Yumian19 | 0.6993 | 0.3007 | 1 | the Yellow River valley, China |
| 2 | ZhongArc-308 | 0.6996 | 0.3004 | 2 | the Yellow River valley, China |
| 2 | ZhongArc-185 | 0.7012 | 0.2988 | 2 | the Yellow River valley, China |
| 2 | Keyi2 | 0.7057 | 0.2943 | 1 | the Yangzi River valley, China |
| 2 | CCRI35 | 0.7092 | 0.2908 | 1 | the Yellow River valley, China |
| 2 | LOC23/757 | 0.7208 | 0.2792 | 2 | Australia |
| 2 | B1319 | 0.7208 | 0.2792 | 3 | the Yangzi River valley, China |
| 2 | ZhongArc-105 | 0.7255 | 0.2745 | 2 | the Yellow River valley, China |
| 2 | PD829 | 0.7288 | 0.2712 | 4 | U.S.A. |
| 2 | DP410B | 0.7303 | 0.2697 | 4 | U.S.A. |
| 2 | M-8124-1159 | 0.7442 | 0.2558 | 4 | U.S.A. |
| 2 | V4017 | 0.753 | 0.247 | 3 | the Yangzi River valley, China |
| 2 | Jimian7 | 0.7552 | 0.2448 | 1 | the Yellow River valley, China |
| 2 | CCRI23 | 0.758 | 0.242 | 1 | the Yellow River valley, China |
| 2 | ZhongARNAuXu | 0.7605 | 0.2395 | 2 | the Yellow River valley, China |
| 2 | CCRI36 | 0.7695 | 0.2305 | 1 | the Yellow River valley, China |
| 2 | Dai16 | 0.7697 | 0.2303 | 4 | U.S.A. |
| 2 | Zhong932906 | 0.7743 | 0.2257 | 2 | the Yellow River valley, China |
| 2 | R808 | 0.7748 | 0.2252 | 3 | the Yellow River valley, China |
| 2 | Yu17-202 | 0.7779 | 0.2221 | 3 | the Yellow River valley, China |
| 2 | Yumian1 | 0.7782 | 0.2218 | 1 | the Yangzi River valley, China |
| 2 | King cotton | 0.7858 | 0.2142 | 4 | U.S.A. |
| 2 | Shan6192 | 0.7897 | 0.2103 | 3 | the Yellow River valley, China |
| 2 | Jifeng197 | 0.7949 | 0.2051 | 1 | the Yellow River valley, China |
| 2 | R-1 | 0.8251 | 0.1749 | 3 | the Yellow River valley, China |
| 2 | Shan401 | 0.8406 | 0.1594 | 3 | the Yellow River valley, China |
| 2 | Arcot-1 | 0.8417 | 0.1583 | 4 | U.S.A. |
| 2 | Yumian9 | 0.8442 | 0.1558 | 1 | the Yellow River valley, China |
| 2 | J02-508 | 0.8466 | 0.1534 | 2 | the Yellow River valley, China |
| 2 | MSCO-12 | 0.847 | 0.153 | 4 | U.S.A. |
| 2 | 601changrong | 0.8543 | 0.1457 | 2 | the Yangzi River valley, China |
| 2 | UZBEK713 | 0.8572 | 0.1428 | 4 | the Uzbek |
| 2 | Xiangkang85-1 | 0.8669 | 0.1331 | 3 | the Yangzi River valley, China |
| 2 | Zhekangnong83-811 | 0.8674 | 0.1326 | 3 | the Yangzi River valley, China |
| 2 | Forster6 | 0.8733 | 0.1267 | 1 | U.S.A. |
| 2 | V4014 | 0.875 | 0.125 | 3 | the Yangzi River valley, China |
| 2 | S-050031 | 0.8754 | 0.1246 | 2 | the Yellow River valley, China |
| 2 | 3120 | 0.8768 | 0.1232 | 3 | the Yangzi River valley, China |
| 2 | Liaomian4 | 0.8881 | 0.1119 | 1 | Northern Specific early maturation, China |
| 2 | ZhongAR683-77 | 0.8907 | 0.1093 | 2 | the Yellow River valley, China |
| 2 | STV2B | 0.8925 | 0.1075 | 4 | U.S.A. |
| 2 | 52-128 | 0.8932 | 0.1068 | 3 | the Yangzi River valley, China |
| 2 | Yumian5 | 0.8961 | 0.1039 | 1 | the Yellow River valley, China |
| 2 | ZhongZi04184 | 0.8965 | 0.1035 | 2 | the Yellow River valley, China |
| 2 | Zhong31-204 | 0.8986 | 0.1014 | 2 | the Yellow River valley, China |
| 2 | ZhongR773-75 | 0.9103 | 0.0897 | 2 | the Yellow River valley, China |
| 2 | Ji91-32 | 0.9116 | 0.0884 | 3 | the Yellow River valley, China |
| 2 | Su08B2-177 | 0.9135 | 0.0865 | 3 | the Yellow River valley, China |
| 2 | UZBEK2287 | 0.9148 | 0.0852 | 4 | the Uzbek |
| 2 | ZhongR773-72 | 0.9152 | 0.0848 | 2 | the Yellow River valley, China |
| 2 | AmD-1 | 0.9314 | 0.0686 | 4 | U.S.A. |
| 2 | ZhongR773-309 | 0.9315 | 0.0685 | 2 | the Yellow River valley, China |
| 2 | PD9363 | 0.9378 | 0.0622 | 4 | U.S.A. |
| 2 | ZhongZi4480 | 0.9389 | 0.0611 | 2 | the Yellow River valley, China |
| 2 | UZBEK901 | 0.9471 | 0.0529 | 4 | the Uzbek |
| 2 | Han8901 | 0.9487 | 0.0513 | 1 | the Yellow River valley, China |
| 2 | PD3249 | 0.9531 | 0.0469 | 4 | U.S.A. |
| 2 | ZhongR773-310 | 0.9569 | 0.0431 | 2 | the Yellow River valley, China |
| 2 | MM-2 | 0.9571 | 0.0429 | 4 | U.S.A. |
| 2 | ZhongR773-314 | 0.9614 | 0.0386 | 2 | the Yellow River valley, China |
| 2 | UZBEK | 0.9623 | 0.0377 | 4 | the Uzbek |
| 2 | Shanmian1 | 0.9637 | 0.0363 | 1 | the Yellow River valley, China |
| 2 | J02-247 | 0.9688 | 0.0312 | 2 | the Yellow River valley, China |
| 2 | Xuzhou1818 | 0.9714 | 0.0286 | 3 | the Yellow River valley, China |
| 2 | KucheT94-6 | 0.9725 | 0.0275 | 3 | Northwestern inland, China |
| 2 | ZhongZi9196 | 0.9879 | 0.0121 | 2 | the Yellow River valley, China |

**^a^** 1 Elite varieties that have been popularly cultivated in China; 2 Germplasm resource lines with outstanding character of yield component or fiber quality; 3 Parent lines used in the breeding program; 4 Non-domestic historical varieties and germplasm resources lines.
