# Supplementary material for: Identification of Associated SSR Markers for Yield Component and Fiber Quality Traits Based on Frame Map and Upland Cotton Collections
Source: PLoS One. 2015 Jan 30;10(1):e0118073. doi: 10.1371/journal.pone.0118073 (PMC4311988; doi:10.1371/journal.pone.0118073)
Supplement: S2 Table — (DOC) [file pone.0118073.s005.doc]

**Table S2.** Frequency distribution of LD (r^2^) of marker pairs in the 241 Upland cotton collections (*p* < 0.05)

|  | Pairs loci | Pairs of LD loci | 0~0.1 | 0.1~0.2 | 0.2~0.4 | 0.4~0.6 | 0.6~0.8 | 0.8~1.0 | Means of r^2^ |
| --- | --- | --- | --- | --- | --- | --- | --- | --- | --- |
| Sum of collinear loci pairs (No.) | 11392 | 2153 | 1281 | 244 | 294 | 145 | 125 | 64 | 0.18 |
|
| Sum of non-collinear loci pairs (No.) | 220381 | 19541 | 18867 | 572 | 71 | 17 | 0 | 14 | 0.03 |
|
| Total (No.) | 231773 | 21694 | 20148 | 816 | 365 | 162 | 125 | 78 | 0.05 |
|
