# Supplementary material for: Identification of Associated SSR Markers for Yield Component and Fiber Quality Traits Based on Frame Map and Upland Cotton Collections
Source: PLoS One. 2015 Jan 30;10(1):e0118073. doi: 10.1371/journal.pone.0118073 (PMC4311988; doi:10.1371/journal.pone.0118073)
Supplement: S3 Table — (DOC) [file pone.0118073.s006.doc]

Table S3. Phenotypic performance of yield and fiber quality traits across four environments
Trait	Environment	Mean	Min	Max	SD	Skewness	Kurtosis	
NB(No.)	E1	14.01 	6.49 	26.75 	3.35 	0.55 	1.00 	
	E2	19.51 	8.64 	32.88 	4.48 	0.15 	-0.19 	
	E3	26.89 	14.47 	40.44 	4.58 	0.13 	0.32 	
	E4	19.24 	10.88 	28.52 	3.37 	0.05 	-0.14 	
BW(g)	E1	4.25 	2.93 	5.47 	0.46 	0.00 	0.28 	
	E2	4.82 	3.06 	6.37 	0.52 	-0.12 	0.88 	
	E3	5.30 	3.99 	6.47 	0.46 	-0.03 	0.04 	
	E4	5.15 	2.94 	6.40 	0.55 	-0.40 	0.68 	
LP (%)	E1	38.86 	28.22 	45.00 	2.95 	-0.45 	0.06 	
	E2	38.36 	29.11 	45.45 	2.96 	-0.32 	-0.07 	
	E3	39.10 	28.17 	46.74 	3.13 	-0.39 	0.38 	
	E4	39.77 	30.06 	46.94 	2.76 	-0.68 	1.06 	
FL(mm)	E1	28.82 	25.21 	33.33 	1.55 	0.41 	0.16 	
	E2	30.94 	26.53 	34.80 	1.25 	0.16 	0.88 	
	E3	29.74 	26.72 	33.47 	1.23 	0.28 	0.28 	
	E4	29.47 	26.38 	32.74 	1.14 	0.18 	0.66 	
FS(cN/tex)	E1	28.15 	24.27 	35.40 	1.61 	0.56 	0.43 	
	E2	29.59 	25.10 	36.30 	1.62 	0.88 	1.50 	
	E3	29.48 	26.53 	35.67 	1.42 	1.10 	1.91 	
	E4	28.61 	25.80 	35.87 	1.31 	0.82 	0.98 	
FM	E1	5.02 	3.94 	5.64 	0.34 	-0.66 	-0.04 	
	E2	4.33 	2.22 	5.22 	0.39 	-0.82 	3.10 	
	E3	5.22 	3.50 	5.79 	0.34 	-1.74 	4.80 	
	E4	4.88 	3.95 	5.66 	0.32 	-0.37 	-0.01 	
