# Supplementary material for: Identification of Associated SSR Markers for Yield Component and Fiber Quality Traits Based on Frame Map and Upland Cotton Collections
Source: PLoS One. 2015 Jan 30;10(1):e0118073. doi: 10.1371/journal.pone.0118073 (PMC4311988; doi:10.1371/journal.pone.0118073)
Supplement: S4 Table — *Significant at the p < 0.0001 level. (DOC) [file pone.0118073.s007.doc]

**Table S4.** Mean squares of the ANOVA for yield and fiber quality traits of 241 collections in four environments

| Trait | Source | df | E1 | E2 | E3 | E4 |
| --- | --- | --- | --- | --- | --- | --- |
| NB | Collection | 240 | 35.34* | 45.81* | 60.03* | 32.78* |
|  | Error | 482 | 12.40 | 18.55 | 18.90 | 12.51 |
| BW | Collection | 240 | 0.91* | 0.55* | 0.89* | 0.86* |
|  | Error | 482 | 0.31 | 0.17 | 0.31 | 0.27 |
| LP | Collection | 240 | 21.75* | 19.59* | 27.06* | 21.34* |
|  | Error | 482 | 3.10 | 2.37 | 2.18 | 1.55 |
| FL | Collection | 240 | 5.94* | 3.54* | 4.44* | 3.79* |
|  | Error | 482 | 1.97 | 0.77 | 0.84 | 0.91 |
| FS | Collection | 240 | 6.09* | 5.61* | 5.86* | 4.98* |
|  | Error | 482 | 1.90 | 1.29 | 1.22 | 1.12 |
| FM | Collection | 240 | 0.27* | 0.32* | 0.33* | 0.31* |
|  | Error | 482 | 0.09 | 0.10 | 0.05 | 0.08 |

*Significant at <0.000l evel.
